# Variant alleles of the CYP1B1 gene are associated with colorectal cancer susceptibility

**DOI:** 10.1186/1471-2407-10-420

**Published:** 2010-08-11

**Authors:** Joanna Trubicka, Ewa Grabowska-Kłujszo, Janina Suchy, Bartłomiej Masojć, Pablo Serrano-Fernandez, Grzegorz Kurzawski, Cezary Cybulski, Bohdan Górski, Tomasz Huzarski, Tomasz Byrski, Jacek Gronwald, Elżbieta Złowocka, Józef Kładny, Zbigniew Banaszkiewicz, Rafał Wiśniowski, Elżbieta Kowalska, Jan Lubinski, Rodney J Scott

**Affiliations:** 1 Department of Genetics and Pathology, International Hereditary Cancer Center, Pomeranian Medical University, Szczecin, Poland; 2 III Department of Surgery, Pomeranian Academy of Medicine, Szczecin, Poland; 3 Department of Surgery, Medical Academy, Bydgoszcz, Poland; 4 Regional Oncology Hospital, Bielsko Biała, Poland; 5 Discipline of Medical Genetics, School of Biomedical Sciences, Faculty of Health, University of Newcastle and the Hunter Medical Research Institute, Newcastle NSW Australia; 6 Children's Cancer Research Group, Hunter Medical Research Institute, John Hunter Hospital, Lookout Road, New Lambton, NSW 2305 Australia

## Abstract

**Background:**

CYP1B1 is a P450 enzyme which is involved in the activation of pro-carcinogens to carcinogens as well as sex hormone metabolism. Because differences in the activity of the enzyme have been correlated with variant alleles of single nucleotide polymorphisms (SNPs), it represents an attractive candidate gene for studies into colorectal cancer susceptibility.

**Methods:**

We genotyped 597 cancer patients and 597controls for three CYP1B1 SNPs, which have previously been shown to be associated with altered enzymatic activity. Using the three SNPs, eight different haplotypes were constructed. The haplotype frequencies were estimated in cases and controls and then compared. The odds ratio for each tumour type, associated with each haplotype was estimated, with reference to the most common haplotype observed in the controls.

**Results:**

The three SNPs rs10012, rs1056827 and rs1056836 alone did not provide any significant evidence of association with colorectal cancer risk. Haplotypes of rs1056827 and rs10012 or rs1056827 and rs1056836 revealed an association with colorectal cancer which was significantly stronger in the homozygous carriers. One haplotype was under represented in the colorectal cancer patient group compared to the control population suggesting a protective effect.

**Conclusion:**

Genetic variants within the CYP1B1 that are associated with altered function appear to influence susceptibility to a colorectal cancer in Poland. Three haplotypes were associated with altered cancer risk; one conferred protection and two were associated with an increased risk of disease. These observations should be confirmed in other populations.

## Background

Adverse interactions between DNA and environmental toxins may be mitigated through a range of biologic defense mechanisms, including DNA repair, cell cycle checkpoint control and xenobiotic clearance enzymes. Xenobiotic clearance is important for the removal of carcinogens and is primarily accomplished by hydroxyl conjugation, involving enzymes in the cytochrome P450 pathway [1]. Cancers of the colon, lung, larynx, kidney and pancreas have been shown to be associated with environmental exposures to various carcinogens [2,3] and polymorphisms in several key enzymes involved in xenobiotic clearance have been linked to the risks of various cancers. One enzyme of particular importance is CYP1B1, which is encoded by a polymorphic gene [3]. A number of polymorphisms in this gene have been shown to affect the activity of the encoded protein [4,5]. Four polymorphisms, occurring at codons rs10012, rs1056827, rs1056836 and rs1800440, all of which result in single amino acid substitutions that result in an altered enzyme activity are of particular interest [5]. CYP1B1 is primarily involved in the hydroxylation of 17β-estradiol at the 2-OH and 4-OH positions, which can then be oxidized to semiquinones and quinines [1]. Both semiquinones and quinones are electrophilic metabolites that can form DNA adducts thereby potentially introducing mutations into the genome [6]. Generation of 2-OH catechol estrogens does not appear to result in deleterious affects whereas the 4-OH catechol derivative has been shown to be associated with an increase in DNA single strand breaks as well as increased nuclear levels of 8-hydroxyguanosine [5,7,8]. Both of these undesirable outcomes alter the risk of malignancy by increasing the likelihood of introducing mutations into the genome and consequently the risk of malignancy.

As part of a major initiative to identify major cancer susceptibility genes for colorectal cancer, we investigated three common variants in the CYP1B1 gene that individually confer a difference in the activity of the encoded protein. All three variants, codon 48G > C (rs10012), codon 119G > T (rs1056827) and 432 G > C (rs1056836) have been shown to result in an increased metabolism of 16a-OH-estradiol [5]. An investigation of three of the CYP1B1 SNP polymorphisms was undertaken to determine whether, alone or in combination, these SNPs were associated with an altered likelihood of disease. A fourth CYP1B1 variant, SNP 453A > G (rs1800440) was not investigated in the present study because it does not occur at sufficiently high frequency to allow a meaningful analysis.

## Methods

### Study subjects

The Polish population is extremely homogeneous and lends itself to population based studies as there are no significant ethnic differences to take into consideration. 597 colorectal cancer patients were enrolled in the study. Enrollment occurred between 1998 and 2006 from 3 cities in Poland (Table 1). Patients were asked to participate during a visit to the outpatient oncology departments of the various participating hospitals affiliated with the Hereditary Cancer Center in Szczecin. To estimate the prevalence of the various CYP1B1 genotypes and haplotypes in the underling Polish population, 597 sex and age-matched controls were invited to participate in the study all of whom resided in the city and surrounds of Szczecin.

**Table 1:** Sites and period of specimen collection for the patients samples

	Colon
Time period of collection of cases	1998-2006
Hospitals where patients were collected	Bielsko-Biała Bydgoszcz Szczecin
Age range of diagnosis	21-92

The study protocol was approved by the ethics committee of the Pomeranian Medical University and each participant signed a consent form for the study prior to enrolment.

### DNA isolation

DNA was extracted from peripheral blood lymphocytes of individuals using the non-enzymatic, rapid method without modification [9].

### Polymorphism Analysis

**Figure 1:**
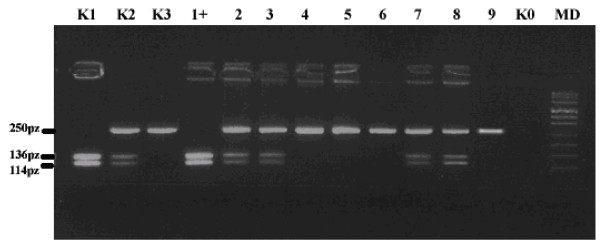
**Representative gel of RFLP-PCR for change 355G > T (A119S)**. **Lanes: K1 **positive control homozygote 355TT, **K2 **positive control heterozygote 355GT, **K3 **positive homozygote 355GG, 1+ homozygote 355TT, **lanes 2,3,7 & 8 **hetereozygote 355 GT, **lanes 4-6 & 9 **homozygote 355 GG, **K0 **negative control, **MD **pUC Mix 8

The A119 S variant was identified by restriction fragment length polymorphism PCR using CTCGTTCGCTCGCCTGGCGC and GAAGTTGCGCATCATGCTGT primers. The PCR reactions was carried out in PTC-200 Peltier DNA Thermal Cycler MJ Research. Detailed experimental PCR conditions are available on request. Amplified DNA was digested with Eam 1105I enzyme and size separated on a 3% agarose gel. The PCR product length, before and after digestion were 136 base pairs (bp) and 114 bp, respectively (see Fig 1) All positive results were verified by a reverse RFLP-PCR using a Pdil enzyme (Fermentas, St Leon Rot, Germany). Finally, a random selection of cases harbouring the GG, GT or TT genotypes were subjected to direct sequencing using the BigDye Terminator Ready Reaction Kit v3.0 (Applied Biosystems, Foster City, CA) and analyzed in an ABI PRISM 377 DNA sequencer (data not shown).

A similar protocol was performed for the 142G > C and 432C > G change using the primer pairs TCCATCCAGCAGACCACGCT and GCCGGACACCACACGGAAG and ATGCGCTTCTCCAGCTTTGT and TATGGAGCACACCTCACCTG, respectively.

**Figure 2:**
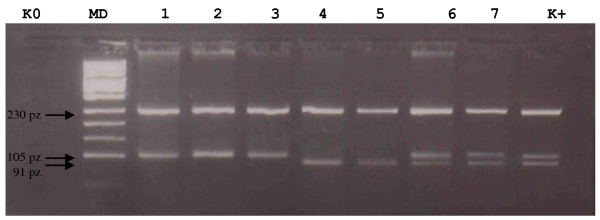
**Representative gel of RFLP-PCR for 142C > G (R48G)**. **Lanes: K0 **negative control, **K+ **positive control (heterozygote 142CG), **MD **pUC Mix 8, **1 **homozygote 142CC, **2 **homozygote 142CC, **3 **homozygote 142CC, **4 **homozygote 142GG, **5 **homozygote 142GG, **6 **heterozygote 142CG, **7 **heterozygote 142CG.

**Figure 3:**
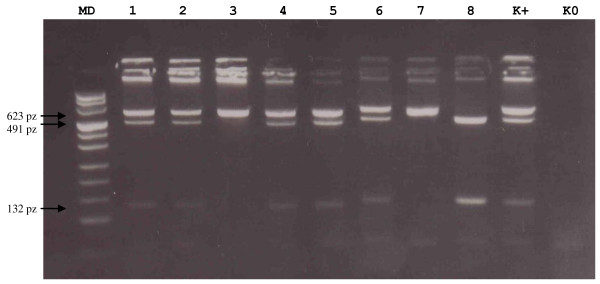
**Representative gel of RFLP-PCR for change *CYP1B1*-V432L (4329G > C)**. **Lanes MD **pUC Mix 8, **K+ **positive control heterozygote 4329 GC (V432L), **K0 **negative control. Labes 1,2, 4-6 heterozygote 4329GC, lanes 3,7 homozygote 4329GG, lane 8 homozygote 4329CC

The 142G > C polymorphism was identified using the restriction enzyme AvaI enzyme (Fermentas). The resultant products were size separated on 4% agarose gels. The uncut product was 335 bp in size and the cut product was predicted to yield 4 fragments of 230,105, 91 and 14 bp in size. Only the 230,105 and 91 base pair fragments were visualized, since the 14 base pair product was too small to be readily detected using this approach (see Fig 2). In addition, randomly selected cases with G/G, C/C and C/G variants were sequenced (as described above) in order to confirm the presence of the R48G change (data not shown). The 432C > G change was identified using a similar protocol but the enzyme used for the RFLP was the OliI restriction endonuclease (Fermentas, St Leon Rot, Germany) which resulted in two fragments of 132 bp and 491 bp in size in samples that contained the C nucleotide (see Fig 3). Randomly selected cases with G/G, C/C and C/G variants were sequenced in order to confirm the presence of the 432G > C change.

### Statistical analysis

The frequencies of the individual genotypes were compared between the cases and controls. Statistical significance of the odds ratios were tested using the chi-squared analysis (with Yates correction for continuity) and Fisher's exact test, where appropriate. Bonferroni correction was applied to take into account multiple testing.

Eight haplotypes could be generated from the three SNPs. The frequencies of each of these were estimated in the cases and controls using the genetic statistical package haplo.stats for R http:// cran.r-project.org/src/contrib/Descriptions/haplo.stats.html as reported by Schaid et al. [10]. The CGG haplotype was the most common and was used as the standard by which the odds ratios were calculated. The association between the presence of a particular haplotype and cancer risk was determined by the haplo.stats software using a score test (haplo.score) included in the package which is designed to detect statistically significant differences in the distribution of the estimated haplotype frequencies. Each of the three SNPs was in Hardy-Weinberg equilibrium in the cases and controls.

Each SNP of the CYP1B1 gene generated three genotypes, including two homozygote states and one heterozygote state. For each SNP, for each site, the odds ratio and 95% confidence intervals were constructed. For these comparisons, the most common allele was considered to be the normal allele and odds ratios were constructed with reference to individuals with two common alleles. The control population for this study was used to determine the relative frequency of the CYP1B1 alleles in the underlying Polish population.

## Results

For each individual SNP genotyped no significant association between it and colorectal cancer was observed (see Table 2) suggesting that disease risk was not influenced by any of the SNPs alone. This suggests that the resultant affect of each of the polymorphisms alone on protein function was, at best, weak. Since each SNP chosen for this investigation has previously been associated with a change of function in the CYP1B1 gene, a genotype analysis was undertaken comparing two SNPs that were in proximity to one another to determine if the combination of two SNPs was more likely to be associated with disease risk. This analysis entailed assessing rs1056827 with rs10012 and rs1056827 with rs1056836 to determine if these combinations were associated with CRC risk. The most significant finding of this analysis was the identification of three genotypes, two of which were associated with an increased risk of disease and one that was protective (see Table 3). The combination of homozygous wild type rs10012 and heterozygous rs1056827 was associated with a significant increase in CRC risk OR 2.4 CI 1.41-4.05; P 0.001. When rs1056827 SNP was present in the homozygous state an even greater effect was observed (OR 7.1 CI 1.61-31.58; P = 0.04).

**Table 2:** Odds ratios for colorectal cancer associated with the three CYP1B1 genotypes

SNP		CASES	CONTROLS	OR	P-VALUE	95%CI
		N = 597	N = 597			
rs10012	CC	261	265	0.9	0.9	0.9-1.1
	GC	266	265	1.0	1.0	0.8-1.2
	GG	70	67	1.0	0.8	0.7-1.5
						
rs1056827	GG	249	277	0.8	0.1	0.6-1.0
	TG	271	259	1.0	0.5	0.9-1.4
	TT	77	61	1.3	0.2	0.9-1.8
						
rs1056836	CC	214	206	1.0	0.7	0.8-1.3
	CG	275	265	1.0	0.6	0.8-1.3
	GG	108	127	0.8	0.9	0.6-1.0

**Table 3:** Analysis of genotypes R48G and A119 S and frequency in colorectal cancer

CYP1B1		CASES n = 597		CONTROL n = 597		STATYSTICAL ANALYSIS	
rs10012	rs1056827	n	Frequency [%]	n	Frequency [%]	OR	p-value
	GG	199	33.3	241	40.4	0.7	0.08*
CC	GT	48	8.0	21	3.5	2.4	0.001*
	TT	14	2.4	2	0.3	7.1	0.004*
	GG	38	6.4	29	4.9	1.3	0.3
CG	GT	216	36.2	233	39.0	0.9	0.3
	TT	12	2.0	4	0.7	3.0	0.1
	GG	12	2.0	6	1.0	2.0	0.2
GG	GT	7	1.2	6	1.0	1.2	1.0
	TT	51	8.54	55	9.2	0.9	0.7

* after Bonferroni correction

When rs1056827 was investigated with rs1056836 a similar trend was forthcoming (Table 4), where homozygous carriers of the rs1056827 SNP were more likely to develop colorectal cancer compared to there wild type counterparts OR 10.2 CI 1.295-79.61; P = 0.09, after Bonferroni correction) in the presence of homozygote carriers of the rs1056836 SNP. In support of this observation, a similar trend for heterozygote carriers of the rs1056836 allele was also observed in the colorectal cancer group (OR 6.1 CI 1.36-27.39; P = 0.12, after Bonferroni correction). Interestingly, individuals who were heterozygous for rs1056836 and 1056827 but wild type for rs10012 were not associated with an increased risk of disease.

**Table 4:** Analysis of genotypes A119 S and V432L and frequency in colorectal cancer

CYP1B1		CASES n = 597		CONTROL n = 597		STATYSTICAL ANALYSIS	
rs1056827	rs1056836	n	Frequency [%]	n	Frequency [%]	OR	p-value
	CC	60	10.05	45	7.5	1.4	0.2
GG	GC	103	17.2	119	20.0	0.8	0.3
	GG	86	14.4	112	18.8	0.7	0.1

	CC	99	16.6	103	17.3	1.0	0.8
GT	GC	160	26.8	143	24.0	1.2	0.3
	GG	12	2.0	14	2.4	0.9	0.8

	CC	55	9.2	58	9.7	0.9	0.8
TT	GC	12	2.0	2	0.3	6.1	0.12#
	GG	10	0.8	1	0.2	10.2	0.09^

**# **before Bonferroni correction 0.015

^ before Bonferroni correction 0.011

In order to determine if there were groups of individuals who may be greater risk by being homozygous for all three SNPs we investigated the combination of homozygote carriers against each other, where the wild type haplotypes were considered to represent population risk (see Table 5). This analysis was restricted as a result of there being too few homozygote carriers available to undertake an exhaustive analysis of disease risk conferred by all possible combinations of genotypes. Notwithstanding, we were able to examine enough patients and controls to determine that homozygous for the following haplotype C-T-G appeared to be significantly at risk of developing colorectal cancer (OR 21.4 CI 1.2-365.56; P = 0.0019). The combination of being homozygote for rs1056827 and rs1056836 SNPs did not appear to be at any increase risk of disease.

**Table 5:** Haplotype analysis of CYP1B1 and colorectal cancer risk

CYP1B1			ALL	
rs10012	rs1056827	rs1056836	OR	p-value
CC	GG	CC	1.0	1.0
		GG	0.7	0.1
	
	TT	CC	0.2	0.5
		GG	21.4	0.0019*

GG	GG	CC	3.0	0.2
		GG	7.0	0.3
	
	TT	CC	0.9	0.7
		GG	0.3	1.0

*After Bonferroni correction.

## Discussion

This study examines the potential influence of CYP1B1 genetic variants alone, and in combination, on colorectal cancer risk. Each of the three SNPs when considered individually appeared not to be associated with disease risk for colorectal cancer. When considered together, the magnitude of the colorectal cancer risk appeared to be consistent yet modest. One previous report of CYP1B1 and CRC risk provided some evidence for an association with disease but this disappeared once a correction was applied for multiple testing [11]. The report also concluded even though no unequivocal findings were identified the role of CYP1B1 in colorectal cancer risk could not be ruled out, especially in light of histopathological evidence indicating that CYP1B1 is over-expressed in colorectal cancers [12].

Several studies have examined the relationship between individual CYP1B1 polymorphisms and cancer risk [12-17]. The strength of the current study is the investigation of three CYP1B1 SNPs in an ethnically-homogeneous population of controls and patients with one of the most frequently observed malignancies in Poland. The effects of the three SNPs appear modest and would not have been identified in smaller studies.

The variants in codons 119 and 432 are associated with different substrate specificities and consequently the catalytic activity of the enzyme [16,17]. There is evidence that the CYP1B1 variants R48G, A119 S and L432V all exhibit greater catalytic 4-hydoxylation activity than the wild type enzyme [8], which may provide a functional clue as to why there appears to be an, albeit small, association with disease risk.

Finally, in studies similar to the one reported herein it would have been improved by including the effects of environmental factors on disease risk. The current study was not intended to address environmental influences on disease risk rather to determine whether or not there is an over-representation of genetic markers in a case compared to a control population thereby providing information on the relative involvement of the genetic marker on disease risk.

In summary, the CYP1B1 variants examined in this study suggest that they contribute to inter-individual differences in cancer risk and are potentially valuable in genetic risk assessment. These findings have potentially important implications for genetic risk assessment and for prevention studies, but it is important that they be confirmed in other colorectal cancer populations prior to introducing them in the clinical setting.

## Conclusion

Genetic variants within the CYP1B1 that are associated with altered function appear to influence susceptibility to a colorectal cancer in Poland. Three haplotypes were associated with altered cancer risk; one conferred protection and two were associated with an increased risk of disease. These observations should be confirmed in other populations.

## Competing interests

The authors declare that they have no competing interests.

## Authors' contributions

JT participated in the design and coordination of the study and carried out the molecular genetic studies, performed the statistical analysis and the manuscript. EGK participated in the collection of samples and carried out the molecular genetic studies. JS participated in the collection of samples. BM performed the statistical analysis. PSF participated in the design of the study and performed the statistical analysis. GK participated in the collection of samples. CC participated in the design of the study. BG participated in the design of the study. TH participated in the collection of samples. TB participated in the collection of samples. JG participated in the collection of samples. EZ carried out the molecular genetic studies. JK participated in the collection of samples. ZB participated in the collection of samples. RW participated in the collection of samples. EK coordinated the preparation of material for the study. JL conceived of the study and participated in its design and coordination. RJS conceived of the study and participated in its design, prepared the manuscript. All authors read and approved the final manuscript.

## Pre-publication history

The pre-publication history for this paper can be accessed here:

http://www.biomedcentral.com/1471-2407/10/420/prepub
